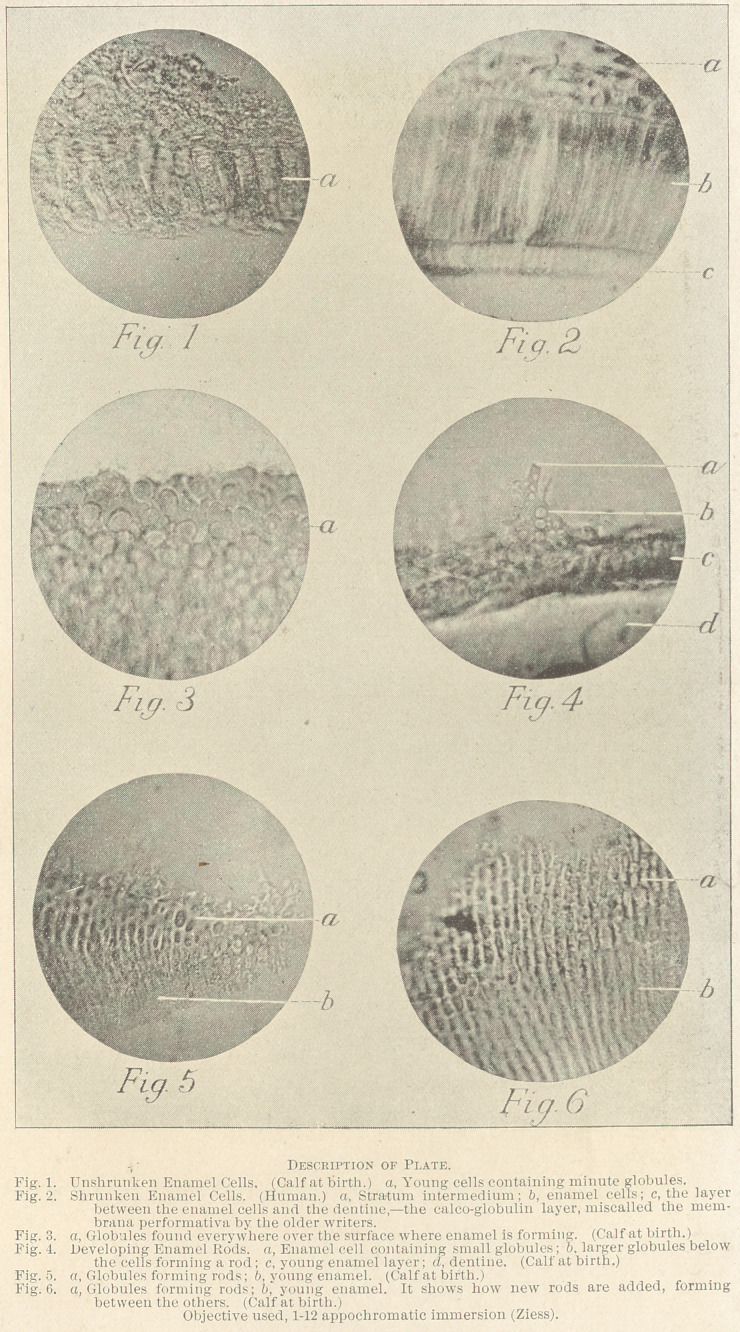# The Formation of Enamel

**Published:** 1891-05

**Authors:** R. R. Andrews


THE
### International Dental Journal.

Vol. XII. May, 1891. No. 5.

### Original Communications.[1]

### THE FORMATION OF ENAMEL.[2]

BY R. R. ANDREWS, D.D.S.

Mr. President, Ladies, and Gentlemen,—In 1888 I had the honor to read a paper before the Odontological Society of Pennsylvania; my subject was "The Development on the Dentine." In my paper I called special attention to the formation of that peculiar layer which is everywhere found between what was fully calcified dentine and the pulp-tissue from which it is formed. It is found in teeth that have been decalcified by the action of weak acid. This issue, which is hyaline in appearance, was, I stated, formed from minute globular bodies that were seen to be in the odontoblasts; they have the appearance of fat-globules, but they are not fat. They are also seen on the surface of the layer, where, by coalescing or merging into each other, they formed large globular masses, and these globular masses coalescing formed the layer of tissue to which Professor Harting has given the name calcoglobulin. It is by further impregnation of lime to become the fully calcified basis substance.

[1] The editor and publishers are not responsible for the views of authors of papers published in this department, nor for any claim to novelty, or otherwise, that may be made by them. No papers will be received for this department that have appeared in any other journal published in the country.

[2] Read at the Union Meeting, Boston, Mass., October 28 to 31, 1890.

I have so far modified my views since reading this paper as to now believe that the odontoblasts, in forming the matrix of the dentine, are only concerned in giving out minute globular bodies, which form the layer of calcoglobulin, not being themselves any part of the matrix. I still hold that the fibres within the tubes are formed from a separate cell deeper in the pulp-tissue, whose processes pass in through the protoplasmic mass of the odontoblasts, and thence into the dentinal tubules of the formed dentine, or, in other words, that the basis substance is formed by a deposit of minute globular bodies, and not by a direct change of the protoplasm of the cells.

This subject proved so interesting to me that I turned my attention to the investigation of the formation and calcification of enamel. My specimens were freshly prepared to show the tissue as near life as possible. My method is somewhat different from those used heretofore. I take the forming teeth from the jaws at or nearly the time of birth, while the tissue is still warm and moist. They are then placed in a one-half of one-per-cent. solution of chromic acid, and this I changed three times daily for three or four days. The edges of the calcified tissue were then found to be sufficiently softened to make a number of thin sections. I first wash the teeth in distilled water, and then place them in a solution of gum-arabic for several hours. Alcohol is used to take out the water, and they are embedded in a preparation of paraffin and lard, which has been poured into a convenient mould, and they are, when cool, ready for section cutting.

The microtome which I use has this advantage over others: the tissue and knife are both under fluid when the sections are cut, and as they are cut they float off and remain in the fluid until they are taken up for examination. I cut until the tissue which has not been decalcified is reached, and each cutting ruins the edge of my knife; but I have the satisfaction of working as near life as I can with my present knowledge. After cutting, the sections are examined, and those which I think are worth keeping are placed in distilled water for a short time to dissolve out the gum, and then are mounted in glycerin jelly. By this simple method I avoid shrinking or shrivelling the tissues, as tissues are when they are kept a long time in acids, in absolute alcohol, oil of cloves, and other reagents, or by the drying processes that prepare them for the beautiful serial sections that are turned out from many of our laboratories, where the Thoma microtome is used.

I regard the generally accepted theory of the conversion of the cells of the internal epithelium of the enamel organ, the ameloblasts, into the rods or fibres of the enamel as an erroneous one, from the fact that I have often observed in developing teeth a folding-in upon itself of that tissue which is to become enamel, which is found between the formed dentine and the cells of the internal epithelium of the enamel organ. This folded layer is undoubtedly a band of uncalcified tissue, and seems to be formed in these folds, that it may be taken up by the growth of the dentine germ during its rapid growth.

I have quite a number of specimens showing the folding-in of this tissue, mostly from pigs' embryos at birth, and this appearance is so marked that it is impossible for me to give any other interpretation.

This peculiar layer has received attention from most of the investigators. Professor Huxley has stated that it is possible to raise a continual sheet of tissue or membrane from the surface of developing enamel. He concludes that this is the original membrana præformativa of the older writers, and that this eventually becomes Nasmyth's membrane. He believes that the enamel was developed without any direct action of the enamel organ, because a membrane separated the two. Tomes believes that this sheet or membrane is produced solely by the destructive action or reagents. Markson believed that it was nothing more than the part of the papilla first calcified.

Dr. Lionel Beale denied the existence of any membrane between the enamel and the enamel-cells. Robin and Magitot offer an explanation of the appearance of such a membrane by stating that the formative pulp is rich in a clear substance of gelatinous consistence; that it is dense towards the surface, where it forms a matrix for the ameloblasts and projects beyond them, so as to look in sections like a sort of varnish, between the enamel and the cells. Being dense near the surface, it may become corrugated and look like a folded or torn membrane.

Frey states that as the calcification of dentine is commencing, the surface of the latter is covered with already hardened, but still short, prisms. Not seldom we encounter appearances as if over these prisms there was superimposed a special cuticle, the so-called membrana præformativa. Such a membrane does not in reality exist, however, and the whole is only a deceptive appearance produced by the youngest layer of enamel which is undergoing development, after the decalcification of the whole from the fully-formed tissue beneath.

Klein says that the distal extremity of the enamel-cells, that is, the one next the dentine, elongates, and this elongation he calls an increment, and tells us that this is directly converted into enamel. He states that the increment of the enamel-cells and the conversion into enamel probably occur successively, and this he thinks to be the cause of the striations across the enamel-rods. He states that the enamel-cells, like all epithelial cells, are separated from one another by a homogeneous interstitial substance, and as he finds this substance between newly-formed enamel-rods, he claims that it is by conversion that one is formed from the other. He says that in the enamel of a developing tooth that interstitial substance is always found to be larger in amount than in the fully-formed organ, and this appearance I have repeatedly noticed myself.

Dr. Sudduth, in his article on amelification, in the *American System of Dentistry*, gives very little if any attention to the existence of this layer found between the calcified and the organic tissue. He speaks of the substance found between the rods of young enamel as a basis substance composed of calcoglobulin; he calls attention to important facts which I, myself, have noticed, and that is, wherever enamel is forming the stellate reticulum has disappeared, aud the stratum intermedium seems directly in contact with the capillaries of the connective tissue without. It seems essential that capillary vessels should thus be in indirect contact with enamel-cells before the processes of calcification can be commenced. Dr. Sudduth gives not a little space in his chapter on amelification to prove that the enamel, which he rightly calls a coat of mail, and shell, are analogous structures.

There has very recently fallen into my hands, translated by a friend, an article by Dr. Graf Spee, from the *Biologisches Centralblatt*, " On the First Processes of the Deposition of the Enamel." In it he calls the minute spherical bodies, which I call calcospherites, " enamel drops." I will quote a part of his most interesting paper:

" A coarsely granular appearance of the enamel-forming cell has been often observed. Annel rightly claims to have seen highly-refractive granules in the body of the cells, and, according to my experience, these granules are regularly to be found in enamel-forming cells. The abundant appearances of the granules at the time of the formation of the enamel and their entire absence at earlier stages, is an indication that the granules are an enamel substance. I call them enamel drops. Following up their future confirms their appearance. I saw the enamel drops appear only in the half of the enamel-cells which is turned towards the pulp, and within this half at first in the end which rests on the dentine; afterwards farther up in the cell, but not quite up to the region of its nucleus. Many of them were so small as to be scarcely measurable. They are almost always spherical.

"Great numbers of them are collected at the periphery, and appear here either to be completely arranged or to fuse together. At any rate, one soon finds that on the dentine or on the already formed enamel layer there are no longer isolated or enamel drops, but a more homogeneous mass. The lower part of the cell contains the larger enamel drops, which merge without sharp boundaries into the substance of the enamel-fibres. This then appears as a part of the enamel-cell in which the originally isolated enamel drops have run together into a continuous mass. The growth of the enamel-rod, once begun, appears to take place by the addition of new enamel drops.

"The product of the enamel-cells first to be found is, therefore, not the chemically definite enamel, but an organic precursor of it, which is, perhaps, horn-like, since even the enamel cap which is not yet impregnated with salts, has a horny appearance. When there is a formation of earthy deposits under the influence of cells, the process is of such a nature that at first an organic production is formed, which in turn has the power of easily forming in soluble compounds with organic salts, and thus become hardened."

This "horn-like" substance which Dr. Graf Spee found, was, I believe, the changed tissue calcoglobulin to which I have already alluded.

It may not be uninteresting at this point to quote from Quain's Anatomy, vol. ii. p. 71, a few words about other tissues formed from globules. In speaking of the formation of elastic fibres, the writer says, "These, as shown by Ranvier, first appear in the form of granules or globules, which subsequently become fused together, end to end, and are not at any time connected with cells. In elastic cartilage the granules or globules make their appearance, it is true, in the immediate neighborhood of the cartilage-cells, but although this renders it probable that the deposition of the globules is influenced by the cells, it does not prove that they are formed by a direct conversion of the cell protoplasm. Indeed, the subsequent extension of these fibres into those parts of the matrix which were previously clear of them, and in which no such direct conversion of cell protoplasm seems possible, it is a strong argument in favor of the deposition hypothesis. The view which supposes that a direct conversion of the protoplasm of the connective-tissue cells takes place into fibres both white and elastic has of late years been widely adopted, but it seems to rest less upon observation than upon a desire to interpret the facts in accordance with the conceptions of Beale and M. Schultze, according to which every part of an organized body consists either of protoplasm (formative matter) or of material which has been protoplasm (formed material), and the idea of a deposition or change occurring outside the cells, in the intercellular substance is excluded. But it is not difficult to show that a formation of fibres may occur in soft substances in the animal organism independently of the direct agency of cells, although the material for such formation may be furnished by cells. Thus, in those calenterate animals in which a low form of connective tissue first makes its appearance, this is distinguished by a total absence of cellular elements, the ground substance being first developed, and the fibres being formed in it. Again, the fibres of the shell membrane of the bird's egg are certainly not formed by the direct conversion of the protoplasm of the cells. although it is probably in matter secreted by those cells, and through their agency, that the deposit occurs in a fibrous form.

To the dental histologist, enamel is, perhaps, the most difficult subject he is called upon to investigate. While there are many who have shown us the coarser morphology of the enamel organ, there are very few who have had anything to say about the finer processes in the deposition of the enamel. Perhaps on account of the difficulties met with, the subject has received insufficient investigation. The ordinary methods of the laboratories do not give us good results with this tissue. It comes to us so shrunken as to be of little use to satisfactorily demonstrate what the minute bodies, miscalled granules, within the substance of the enamel-cells really are. The shrinking of the cells is caused by the reagents which have been used in their preparation. (See Fig. 2.)

The so-called calcareous granules are minute calcospherites,—they are most spherical. The *nucleus* of the enamel-cell is not always to be found farthest from the calcifying tissue, as authorities inform us. I have sections showing it midway between the two ends of the cell, some being near the layer next the forming enamel, surrounded by very many minute glistening bodies. Between the cell and the calcified material is found the layer of calcoglobulin. (See Fig 2.) It is of the utmost significance to us, because within its substance the first formation of the rods of the enamel takes place. Huxley was right in crediting it with the importance he gave it, but wrong in calling it a membrane.

Fig. 1

Fig. 2

Fig. 3

Fig. 4

Fig. 5

Fig. 6

It is a tissue formed by the globular contents of the enamel-cells, with a portion of the cell protoplasm, a cement substance. In it the globules are arranged in columns and become the rods. (See Fig. 4.) This is the point I would emphasize, showing, as I believe it does, the methods of nature in forming rods, independent of the cells themselves. By a further process, these globules or masses become calcified, and a part of the already calcified rods. (See Figs. 5 and 6.) I have studied this layer from several hundred sections, in studying the formation of dentine as well as of the enamel.

In the dentine we have a formative pulp, full of nerve-bundles, fibres, and capillaries, and, as a consequence, its calcification gives us a matrix full of organic tissue. In the enamel organ there is an almost entire absence of vessels. Its calcification gives us a matrix almost without organic tissue. Enamel in this early age presents the best possible condition to note the presence of a reticulum of living matter if any were present; but with the best objectives at my command, and with all the care that I could give to the subject, I have failed to find the appearance of anything of the kind.

In tearing off portions of the layer with needles, I have found the uncovered calcified matrix to have everywhere over its surface myriads of globular forms (see Fig. 3) resting in a semifluid, the so-called protoplasmic substance. The cells of the internal epithelium of the enamel organ at the time when calcification is commencing are, if they have been carefully prepared as I have already said, found to be full of these minute globules which I believe to be calcospherites. (See Fig. 1.) These are given out from the cell into the layer continually, forming, by coalescing, larger globules or masses which will become the rods, and are surrounded by this protoplasmic substance which is to be the cement substance between the rods; the cells of the stratum intermedium above are seen to be in direct contact with the embryonic connective tissue, the stellate reticulum having disappeared from that part.

My sections having been made extremely thin, were not stained, and the photographs may not show with all the clearness I could wish, but I believe they will give an outline, at least, of what I have referred to. Some of the sections illustrate my point so clearly as to be almost diagrammatic (see Fig. 4); other sections will show that the protoplasmic substance in which the globules or masses are to be seen is forming the cement substance between the young rods. (See Figs. 5 and 6.) Tissues showing these various transitions are mostly from the teeth of calves at birth, some from human embryos, fifth month, and others from pigs at birth. In making sections of tissue which is only partially decalcified, I have been able to obtain some pictures of young enamel almost as we see it in ground sections. In the rods of these sections it is possible to mark out the outlines of the globules, or masses, from which I believe they are formed. (See Fig. 6.)

These globules themselves, before complete decalcification has taken place, consist of that substance which Professor Harting has called calcoglobulin. I do not think that the substance between the rods is to be considered this tissue, although Professor Sudduth, of Minneapolis, in his article on amelification, gives it this name, which should be applied only to the tissues which are formed from the globules. It may be of interest in connection with this subject to repeat here a very brief description of the experiments of Professor Harting and Mr. Rainie on the action of certain lime salts on albumen. These investigators claim that the experiments give us an explanation of the methods of the calcifying processes of the osseous tissues.

Mr. Rainie found that if carbonate of lime be slowly added to a thick solution of albumen, the resultant salt is in the form of globules laminated in structure like tiny onions; the globules in contact become agglomerated into a certain laminated mass, appearing as if the laminæ in immediate opposition were blended with one another. The globular masses, at one time of mulberry-like form, lose the individuality of their constituent smaller globules, and become smoothed down into a single mass. Mr. Rainie suggests, as an explanation of the laminated structure, that the smaller masses have accumulated into concentric layers which have subsequently coalesced, and in the substitution of the globular for the crystalline form in the salt of lime when in contact with albumen he claims to find a satisfactory explanation of the development of bone, teeth, and shells.

Professor Harting has shown that the albumen left behind, after the treatment of these globules with acid, is no longer ordinary albumen. It is profoundly modified, and has become exceedingly resistant to the action of acids, resembling chitine, the substance of which the hard skins of insects consist, rather than any other body. The small and onion-shaped globular bodies he has named calcospherites, and the layer caused by the coalescing of these, calcoglobulin, as it appears that the lime is held in some sort of chemical combination; for the last traces of lime are retained very obstinately when calcoglobulin is submitted to the action of acids in the same manner as does that layer which is found everywhere on the border-land of calcification between the carefully calcified and the formative tissues.

I would add, then, in conclusion, that I believe,—

*First.* That the cells of the internal epithelium of the enamel organ, the ameloblasts, contain in the part nearest the calcifying tissue large numbers of minute glistening bodies, which have been misnamed granules, but which are really calcospherites. (Fig. 1.)

*Second.* That these minute globules are given out from the enamel-cell into a protoplasmic substance which is on the surface of the first formed layer of dentine, or, if enamel is already formed, on the formed enamel; that here they coalesce and form larger globules or irregular-shaped masses. (Figs. 4 and 5.)

*Third.* That in this condition they form a layer of calcoglobulin. The globules or masses are arranged in columns, independent of the enamel cell. (Fig. 4.)

*Fourth.* That this layer is really that which the older writers called the membrana præformativa.

*Fifth.* That the forming rods in this layer calcify and become part of those already calcified, the so-called protoplasmic substance surrounding them becoming the cement substance, as before stated. (Fig. 6.)

[The paper was illustrated by thirty-three photo-micrographs of sections of growing enamel.]

## Figures and Tables

**Fig. 1 Fig. 2 Fig. 3 Fig. 4 Fig. 5 Fig. 6 f1:**